# Global health economics: A complex field with few unequivocal answers

**DOI:** 10.7189/jogh.13.01005

**Published:** 2023-12-20

**Authors:** Igor Rudan

**Affiliations:** Centre for Global Health, Usher Institute, The University of Edinburgh, Edinburgh, UK

To understand the biggest health challenges in the world today, we need to go back at least a hundred years. A century ago, among every hundred people who died on the Earth, about eighty would die from infectious diseases – perhaps even more. In the 21^st^ century, only twenty out of a hundred people or fewer were dying from infectious diseases before the COVID-19 pandemic. This decrease in the past hundred years presents a remarkable health transition in the human population. For hundreds of thousands of years before, infectious diseases dominated as a cause of death. A century ago, even in the most developed countries, the average expected lifespan was about fifty years. Today, it can reach 80, and even 85 years in some high-income countries. A major consequence of this health transition is that the entire human population numbered less than two billion about a hundred years ago, and now there are about eight billion people in the world.

So, by curbing infectious diseases – our oldest and most enduring enemy – we have increased the human population size four-fold and almost doubled the average human lifespan. Antibiotics were introduced for widespread use in the 1940s, followed by vaccines in the 1960s, all the while followed by sustained economic development in the background. A hundred years ago, not a single country in the world could produce ten thousand dollars of gross national income per capita. Today, there are countries that produce seventy thousand dollars per capita or more. This huge economic development resulted in the construction of infrastructure, water purification, better nutrition, and sanitation. Along with antibiotics and vaccines, economic development is another important reason why we live in better health today.

Understanding this sequence of events can help us determine what are today's biggest health priorities. In the poorest countries and in contexts of humanitarian or refugee crises, infectious diseases remain the greatest problem. In countries that have reached a middle level of development, especially in large ones like China, India, or Brazil, there is an ongoing explosion of chronic non-communicable diseases, necessitating the need for their prevention and treatment. Economic costs of these diseases are now starting to affect further development. In the most developed countries, where infectious diseases are curbed and chronic non-communicable diseases controlled, some new challenges are taking center stage, such as the diseases of very old age – which we still do not understand well enough. These include, for example, dementias, i.e. diseases of memory loss, and their subtype – Alzheimer’s disease, which have already become a large problem in the most developed countries, affecting people over the age of 85 in substantial proportion and becoming a major economic burden [1].

## WHAT ARE THE INVESTMENT OPTIONS IN GLOBAL HEALTH?

In 1993, the World Bank published its widely cited report “Investing in Health” [2]. This publication made it quite clear how wealth affects health, and also how health affects wealth – both at the individual level and at the population level. A clear link between health, poverty, and economy was established, where poor population health was no longer thought of as an “additional drain” of national resources, but also an important cause of poverty and weak national economy. The report also concluded that “increasing funding for battling diseases in poor countries (then estimated at a mere $41 per person each year – 1/30^th^ what was spent in rich countries) would not only reduce the burden of disease but also dramatically improve the economies” [2]. The report started an era in global health economics that was dominated by so-called cost-effectiveness analysis (CEA), which suggested that we should start measuring how much is being invested in health and how much of the existing burden of sub-optimal health is being reduced at the population level. Investments in health could start being traced, documented, and measured, but measuring population health itself was a bigger challenge. This led to the conception of the field of global health metrics.

First, it was important to define health, so that departures from the “perfect health” status at the population level could be measured. The World Health Organization's (WHO) definition of health was proposed back in 1948 and has not been amended since: “Health is a state of complete physical, mental and social well-being and not merely the absence of disease or infirmity” [3]. An important feature of the WHO’s definition is that it identifies the “burden” of sub-optimal health that needs to be reduced as both the active disease and the missed potential to full health. So, there are two components of “burden of disease and infirmity” that could serve as a target for investments in health to improve the present situation.

In doing so and achieving better population health, financial resources can be thought of as “energy” that is external to health system, but that can be applied to remove the burden of poor health. As any other energy resource, it would ideally need to be used in the most effective way, rather than “wasted” along the way. Keeping in mind the WHO’s definition, funds can be targeted directly at the problem, e.g. to remove an already active disease in the population through treatment, or be invested to address missed potential for better health, i.e. to improve current state of population health. Both of those goals can be achieved through funding healthcare and health interventions aimed at treatment or improved health.

Whenever the burden of disease and disability is reduced through such investments, the main determinants of diseases – such as the leading risk factors that persist in the population – will inevitably continue to re-introduce the diseases and poor health. Therefore, money can also be targeted at those key determinants of disease, to prevent the emergence of disease or missed potential. This is usually better value-for-money, as it may only be needed once rather than on an annual basis, if the positive change can then be sustained. The implication of recognising this opportunity is that investments in prevention of disease and missed potential for health and investments in their treatment will need to be balanced. Within the latter investment option, further balance will need to be found between investments in treating active disease and fulfilling any missed health potential.

Clearly, financial investments can improve population health through:

1. Investments in healthcare (e.g. building health facilities and purchasing equipment, training health workers, funding available health interventions for prevention or treatment, and delivering the interventions to those who would benefit most);
2. Investments in development assistance for health (e.g. investments in capacity to deliver available interventions, such as supporting health systems or human resources; investments into improved uptake of interventions, such as education on care-seeking; and investments in broader development assistance for health, to improve local contexts – e.g. sanitation, water, roads, education – and to prevent diseases or missed potential for health);
3. Investments in health research: this is yet another possible investment category that can improve population health by allowing health policy makers to better understand the burden of sub-optimal health and its key components, main risk factors, their roles and levels of exposure to risk factors; also, the effectiveness of various interventions (“description” type of health research); health systems and health policies (“delivery”); to improve existing interventions (“development”); and to develop new interventions (“discovery”).

The first option, investing in healthcare and health interventions, is focused mainly on removing the burden of disease and disability by treating those with diseases and making them healthier again. The second option, investing in development assistance for health, is focused mainly on removing the burden by reducing the exposure to risk factors and preventing them from getting a disease in the first place. This leaves a special place for vaccines, as they are a licensed health intervention which also has some attributes of development assistance for health. The third option, investing in health research, is quite complex, as it includes multiple investors and offers a different return on investment in health research. Government agencies, universities, and publicly funded research institutes will invest taxpayers’ money into efforts that should hope to generate new knowledge that will improve health of the entire population. Industry, which also invests heavily in health research (especially big pharmaceutical companies and the biotech industry), may see the increase in their profits and the value of their companies as the main desired return of their investments into health research. Not-for-profit philanthropies and charities may have their own agendas and championed causes, which may not follow the actual needs in the population.

## WHO ARE THE MAIN INVESTORS IN GLOBAL HEALTH?

Aside from governments, which can invest taxpayers’ money in all three options in various proportions (depending on the national and local contexts and the key needs), the industry-based stakeholders (e.g. “big pharma” and “BioTech”), and not-for-profit philanthropies, there is a wide spectrum of other investors who are particularly focused on health problems in low- and middle-income countries, where most of the global burden of disease and disability actually occurs. There are so-called “multilateral organisations”, such as the WHO, The World Bank, United Nations Children’s Fund (UNICEF), Global Alliance for Vaccines and Immunization (GAVI), The Global Fund (for malaria, tuberculosis and acquired immunodeficiency syndrome (AIDS)), UN AIDS, Save the Children Fund in the UK and US, Institut Pasteur, African and Asian Development Banks, Inter-American Development Bank, and others.

There is a special class of investors – mainly in the second option (development assistance for health) – which are called “bilateral organisations”. This is financial aid that is given from taxpayer’s money of the wealthy countries, through decisions of their governments, to much poorer countries. This kind of investing in health can often be used by wealthier countries to enhance their political and economic influence in poorer countries, so the recipient countries can always be quite diverse. Examples of these agencies are the Australian Agency For International Development (AusAID) in Australia, the Canadian International Development Agency (CIDA) in Canada, the United States Agency for International Development (USAID) in the USA, the Department of International Development (DFID) in the United Kingdom, the Norwegian Agency for Development Cooperation (NORAD) in Norway, the Swedish International Development Cooperation Agency (SIDA) in Sweden, the Netherlands Development Cooperation, the GIZ in Germany, the Danish International Development Agency (DANIDA) in Denmark, the French Aid agency (AFD) in France, the Irish Aid in Ireland, and others.

The main investors in health research are the governments (e.g. National Institutes of Health in the USA, the European Commission (EU FP7), and the Medical Research Council and National Institute for Health Research (NIHR) in the UK), The Bill and Melinda Gates Foundation (BMGF), The Wellcome Trust in the UK, the pharmaceutical and biotech industries, but also the growing number of both governmental and non-governmental organisations and private partners. Their relationships can be quite complex. While leading health researchers place high value on answerability, effectiveness, and potential impact on the burden of disease and disability of their research, as well as on its deliverability and the potential effect on equity, investors in health research may have different priorities. Donors can place higher value on clarity and specificity of research ideas, value for money, novelty of research, international competitiveness of the research teams, linkages to broader societal issues, complementarity with other political priorities, and long-term strategic investments.

Some donors may be particularly interested in potential for forming partnerships between researchers and industry to increase the translation of findings and their application. Public-private partnerships have been flourishing in the field of global health in the 21^st^ century. Governmental agencies are also interested in safety and equity lenses and whether implementation of research results would be widening the gaps that are already present in the society. Ministries and international organisations can be mainly interested in deliverability, affordability, and sustainability of the resulting interventions, and in local and national research capacities to carry out the proposed research ideas. Industrial donors are often motivated by opportunities to generate patent-protected intellectual property and translate research results into commercial products. Finally, not-for-profit organisations may be primarily interested in increased media attention for their leading agenda and focus.

## HOW DO INVESTORS IN GLOBAL HEALTH CHOOSE INVESTMENT PRIORITIES?

Ideally, all these stakeholders need tools or methods that review and assess the available evidence for quality, and then use it to formulate investment policies. There is a number of such tools and methods being used today by the funders. One of the first reviews of those tools was performed in 2010 [4]. In this issue of the Journal of Global Health, we are presenting a paper that reviews the development, implementation, and evaluation of one of the most impactful of such tools, called the Equitable Impact Sensitive Tool (EQUIST). Between 1993 and 2012, the principle of CEA underlay most decisions in (global) health economics. Since then, equity became another important principle for consideration, and EQUIST enabled the studying of both cost-effectiveness and equity impact of the proposed investment options in population health, particularly in low- and middle-income countries [5].

The field of global health economics has developed into a branch of science where knowledge about public health, clinical medicine, economics and finance, humanities, political and social sciences, mathematics and statistics, and finally ethics, are all interwoven in a multidisciplinary manner. This is why it can rarely provide any simple answers to the posed questions. All of these approaches are important in health economics and need to be considered – and often some more, depending on a specific question. Global health economics requires a multidisciplinary, multidimensional, systemic thinking, with a large number of more or less independent parameters that need to be considered and harmonised simultaneously, to obtain the best possible answers.

To illustrate why global health economics is so complex, let us start from the postulate that every person today would probably not object to living 100 years in full health, and then dying suddenly and painlessly. That is what humans expect from their lives today – in the best case. If something worse than that scenario happens, they would be unlikely to object to receiving some care and assistance. What would then be the first priority for the state, which collects taxes from the income of its citizens in many countries to then take care of their health? Obviously, any diseases or other causes that can substantially shorten life. It is not the same whether life is shortened at 95 years of age, or in early youth due to traffic accident or gang violence, or during working life because of haematological cancer or neurological disease. Obviously, this is the first level of priority: trying to assist as many citizens as possible to avoid dying before very old age.

The second level of prioritisation could then relate to how suddenly people die prematurely from various diseases. A disease that torments people for years and reduces their quality of life dramatically before an earlier-than-expected death is likely to be a bigger investment priority that a disease that leads to sudden death at the similar age, but without previous symptoms. The third level of prioritisation might consider how common are those diseases among the population. Those that lead to death in a larger number of people will clearly be a higher priority than those that do so in a smaller number of people.

After these quite self-explanatory criteria, some non-medical ones also need to enter decision-making. Another level of prioritisation should consider the financial cost of the existing intervention against these diseases or relevant care. The diseases that are common and impose a large mortality and morbidity burden on the population, and at the same time are cheap to treat, would surely be seen as the highest priority to address. Rare diseases that do not cause severe long-term morbidity but are expensive to treat will be the lowest priority, which can be easily understood. However, the problem in practice is that there are few such extreme examples. Most diseases are somewhere in the middle – that is, either rare, but with very severe symptoms and with very expensive treatments, or common, but with less severe symptoms. How does one prioritise the investments then?

Besides all the diseases and causes that can lead to death, there are also those that affect patients’ quality of life very negatively, but do not lead to death – yet, the patients still expect to be cared for. These include, for example, rheumatic diseases or chronic infection of the bladder. Where do they come in the order of priority? But it gets even more complex, because there are at least a few more important dimensions to consider.

Namely, some diseases primarily affect the poor in society, and others primarily the wealthy. If the policymakers prioritise investments in diseases that affect primarily the wealthy, it is possible to expect strong negative emotional reactions from many people. For policymakers, this will then become quite important, because they may lose many voters between the two elections. Investing in population health can then become quite political. Consequently, the media would likely take an interest and get involved in the process of prioritisation, too.

There are further layers of complexity. Prioritisation criteria mentioned so far relate to investments that could solve problems among those who are already of impaired health. However, money can also be spent on prevention, with the possibility of preventing several diseases at once by influencing a common risk factor. All too often, this is not valued enough in the society because the results are not easily showcased. It is easier to win support of the people when a new diagnostic or treatment device is purchased for the nearby hospital, or when help is provided to someone who is gravely ill. One of key challenges in global health economics is to explain and educate large parts of the population on the value of prevention, because it can help lot more people and it can be financially very efficient and effective.

Besides prevention, investment can also be made in health research, where new interventions can be created that could be better than the currently available ones. This adds an entirely new layer of complexity, because investing in research that can create a new intervention and then deploying it could – at some point in time – be more cost-effective and equitable than supporting the currently leading intervention. It is very difficult to account for this in all priority-setting tools based on evidence, because investments with research are often associated with uncertainty in outcomes.

With all this complexity already present, governments will need to consider that the entire budget for health within a nation, which can be spent in all these different ways, also needs to be reconciled with the budget for other needs of society. Thinking that it would be best to simply invest everything in education and healthcare so that the population could be educated and healthy would be very naive. If the population of a neighbouring nation is quite sick and uneducated, but their leaders invest a lot in arming that country with weapons, then the other neighbouring countries, too, will need to join the arms race, because their general health and education will not help to prevent a possible attack. This is an extreme example, simply to illustrate how difficult it really is to balance the health budget with other societal needs. Global health economics is indeed a highly complex field in its essence, and there are rarely unequivocal answers.

## HOW CAN EVIDENCE BE USED TO IMPROVE INVESTMENT PRIORITISATION IN GLOBAL HEALTH?

With all active and preventable population health problems on one side, and the money that could be used to reduce them on the other, every funding invested in improving health is valuable, and it would be desirable to ensure the greatest possible benefit from each investment. The EQUIST tool, described in this issue of the Journal of Global Health [5], has been used in many countries to develop their “investment case studies” for the Global Financing Facility (GFF), which is hosted at the World Bank. It can be used to prioritise health interventions at various population levels and socio-economic strata. It assists policymakers to understand the “architecture”, i.e. the foundation of population health problems in a certain area and in a population of interest. Then, based on understanding of that foundation – which is likely to differ in different contexts – the EQUIST tool explains which interventions will achieve the best ratio of gains versus investment, i.e. the greatest benefit, while simultaneously positively impacting equity in the population.

EQUIST was developed as an attempt to provide answers to seemingly impossible questions in global health, and to add the dimension of equity to that of cost-effectiveness. Let us say that US$1 million is available to some country as health aid. This could save the lives of 1000 children in the capital of the country, or 800 children somewhere at the periphery, in the poorest part of the nation. Why is it that more deaths can be averted in the capital than at the periphery? Because it costs much more to reach the poorest than to, for example, vaccinate or give antibiotics to those who are easily accessible. In such a case, what should the policymakers do? Can they justify failing to save an additional 200 children's lives in the capital to make political gains by reducing inequalities in society, and helping the poorest children? Can they risk the negative sentiments in the society if this aid is really spent on the wealthiest quintile, even if that is perfectly justifiable by the cost-effectiveness analysis? This is, in essence, a truly difficult problem to which cannot be answered satisfactorily, which demonstrates the complexity of this field. Yet, in the review of 12 years of EQUIST’s implementation, the authors show how this can still be done in an evidence-based way.

Another complex question is how to prioritise investments in health research. The so-called Child Health and Nutrition Research Initiative (CHNRI) method has managed to address many seemingly intractable challenges related to that challenge [6]. It thus achieved broad global application, with well over a hundred published examples of its use in setting research priorities at the global and national level. However, even with these helpful methods, which are rational, evidence-based, transparent, replicable, and democratic, we still cannot answer all questions in global health economics – some will remain too difficult and complex.

## WHAT INNOVATIVE APPROACHES ARE PREFERRED BY THE NON-GOVERNMENTAL INVESTORS?

A new trend among the world's billionaires emerged in the 21^st^ century – they started using their money to solve the health and development problems of the poorest. In other words, the top of the global socio-economic pyramid began to invest in the needs of its bottom. Some of them are now trying to use entirely innovative approaches to solve problems in global health. These are the people who have been extremely successful in the business sector.

Before their entry to the field of global health and development, the basic approach promoted by the WHO was a planned, state-driven activity toward achieving universal healthcare as part of the broader social contract between the state and its citizens. It was designed and implemented by experts from the public sector and academic community. This approach was “horizontal” in its nature, aiming to cover all citizens with healthcare, providing them not only access to care, but also delivering the same standard and quality of healthcare to everyone as much as realistically possible.

Although grounded in good science and evidence, that approach can sometimes feel terribly slow, laborious, and undermined by the migration of health workers – who are the strength of the health system – from poorer to richer countries. This outflow is happening worlwide, weakening health systems in low resource settings where they are most needed. This quickly becomes a serious problem, because it is challenging to educate health workers in the poorest countries.

Billionaire philanthropists are exploring whether they can bring a corporate style of thinking into this complex field, sometimes leading to a clash of key concepts. Instead of the “horizontal” approach, they may sometimes prefer a “vertical” approach. This approach suggests defining major problems, and then gathering the best teams in the world to solve them one by one, rather trying to improve the situation across all diseases. So, they may consider malaria, tuberculosis, or human immunodeficiency virus (HIV) or AIDS in isolation from other global health priorities. This, of course, would not help or satisfy everyone, but for those suffering from specific diseases this approach can be very effective and accelerate progress.

The “vertical” models may begin to seem significantly more efficient than the state-driven, “horizontal” approaches up to a certain extent. However, the potential for help that could be provided through the former approach will be exhausted when the solutions can no longer reach further people in need, because there is no access enabled. At that point, the value of the “horizontal” approach becomes apparent again – however, both approaches are clearly needed.

In global health economics, we need to consider how best to combine these two approaches to reach the so-called “last billion” or “the bottom billion”, where the highest number of deaths and illnesses in the world are concentrated. Those living in an incredibly underprivileged and low-resource conditions in poor city favelas, remote jungles and deserts, those misplaced by wars or natural disasters, or working illegally in the fast-growing cities of middle-income countries where they moved from the countryside, are very difficult to reach and provide with healthcare – this remains the challenge for the field of global health economics.

## WHAT ARE THE MAIN INSIGHTS FROM THE USE OF EVIDENCE-BASED TOOLS IN GLOBAL HEALTH ECONOMICS?

Generally, in most human activities, privatisation and competition lead to price reduction and an increase in quality for users. An interesting characteristic of (global) health economics is that healthcare proves to be quite resistant to those approaches, which otherwise work well in the private sector. Countries that serve as examples of considerable state intervention and planned approach first look at how many new or recurring cases of various diseases or health-related needs they can expect in their population each year. These numbers can be relatively easily predicted with quality disease registers and analysis of time trends. Then, their experts can assess how much the state is able to allocate for healthcare, depending on its economic development, with the aim to offer a certain level of healthcare to everyone.

Next, they can compare the evidence of the burden of health issues and the affordable investments to provide everyone with the same “basket” of standard healthcare, which should ideally be of the same quality, too. However, this also means that no one would be able to get more than that “basket” - unless they pay extra. There is hardly any amount of money that a national healthcare system would not be able to spend, so the need for rationing is immense. Healthcare budgets could easily grow to encompass a substantial proportion of the entire national budget, while people will still be left dissatisfied. Therefore, some countries simply take the approach where they decide that all residents will receive a certain care in the same way, and if someone wants more, then they have to pay for it themselves.

At the other end of the spectrum are countries that almost blindly believe in a liberal approach to private ownership and market competition and their ability to solve all societal needs. Such countries usually have a predominant cultural belief that the rights of an individual are beyond the rights of the community, while the countries from the former example believe in the opposite – that the rights of the entire community are beyond those of any individual. When a culturally preferred individualism is paired with liberalisation of financing of healthcare, the demand for better doctors will increase. Consequently, they can start charging more for their services. The same is with pharmaceutical products - increased demand leads to higher prices, which is the case with all useful technological solutions and devices in medicine. In the end, only some can afford the best care and the most effective medicines after the price increase due to increased demand. The responsibility for one's own health gets transferred from the state to the people themselves.

Although this surely provides incentives for the increased quality of care, many people who fall into difficult life situations will not be able to afford health insurance, leaving a worrying percentage of the population uninsured and without access to quality care. In the same country, there will also be people who are extremely wealthy and have a remarkable health protection, leading to a dramatic stratification of people in terms of their access to healthcare. In these contexts, the life expectancy can become shorter as many as 10-15 years in comparison to what could be achieved with the invested resources if their distribution was more inclusive. Those healthcare systems tend to become inefficient, with high costs and suboptimal health outcomes. Significantly better results for the entire population are achieved with state regulation and carefully planned approaches which provide universal healthcare of comparable quality to all citizens – or getting as close to that ideal as realistically possible. In this, solutions can always be found where investments can be prioritised in a way that is both cost-effective and equitable in the population.
